# Pseudomyxoma Peritonei With Suspected Gastroesophageal Junction Mass: A Rare and Atypical Presentation

**DOI:** 10.7759/cureus.89220

**Published:** 2025-08-01

**Authors:** Adarsh Jawahar Janard, Anu Mary Jackson, Sunil Mathew, Soja Thajudeen, Sameena Tabassum

**Affiliations:** 1 Internal Medicine, Pushpagiri Institute of Medical Sciences and Research Centre, Thiruvalla, IND; 2 Urology, Pushpagiri Institute of Medical Sciences and Research Centre, Thiruvalla, IND; 3 Paediatrics, All India Institute of Medical Sciences, Mangalagiri, Mangalagiri, IND

**Keywords:** diagnostic challenge, elderly female, gastroesophageal junction mass, mucinous ascites, pseudomyxoma peritonei

## Abstract

Pseudomyxoma peritonei (PMP) is a rare clinical entity characterized by the accumulation of mucinous ascites and peritoneal implants, most commonly originating from appendiceal or ovarian neoplasms. Its diagnosis is often delayed due to vague and nonspecific symptoms. We report the case of a 75-year-old female who presented with diffuse abdominal pain, melena, and significant weight loss. Clinical evaluation revealed ascites, and imaging suggested a possible gastroesophageal (GE) malignancy. Ascitic fluid analysis was inconclusive. Due to the markedly distended abdomen and unclear primary pathology, a diagnostic laparotomy was performed. Intraoperatively, mucinous ascites and widespread peritoneal deposits were noted without an identifiable primary tumor. Histopathological analysis confirmed PMP.

MRI and contrast-enhanced CT (CECT) are valuable tools in the diagnosis of PMP, with characteristic features such as visceral scalloping aiding differentiation from other ascitic conditions. Cytoreductive surgery (CRS) with hyperthermic intraperitoneal chemotherapy (HIPEC) remains the gold standard for treatment. However, in this case, the patient’s condition rapidly deteriorated, and she succumbed to multiorgan dysfunction before definitive therapy could be initiated. This report underscores the importance of early suspicion, timely surgical intervention, and referral to specialized centers in managing atypical presentations of PMP.

## Introduction

Pseudomyxoma peritonei (PMP), also known as abdomino-peritoneal mucinous carcinoma (APM), is characterized by mucinous deposits on the peritoneum and extensive intra-abdominal gelatinous ascites. It is most commonly associated with mucin-producing tumors originating from the appendix, stomach, intestine, pancreas, or ovaries. In certain cases, however, a definitive primary source remains unidentified, complicating the diagnostic process [1]. The clinical manifestations range from constitutional symptoms, such as anorexia and fatigue, to abdominal pain and distension due to ascites. These features often mimic other intra-abdominal pathologies, contributing to diagnostic delays.

A hallmark of PMP is the presence of mucinous peritoneal deposits, which are typically confirmed through histopathological examination. Imaging modalities, particularly MRI and contrast-enhanced CT (CECT), aid in the detection of mucinous accumulations and evaluation of disease spread [2,3]. When the primary tumor cannot be identified, diagnosis becomes more challenging. A comprehensive approach, including tumor marker evaluation, cytological analysis, and exploratory laparotomy, is often necessary for accurate assessment [4]. Classification systems, such as the Peritoneal Cancer Index (PCI), are useful for staging the disease and guiding management. Cytoreductive surgery (CRS) combined with hyperthermic intraperitoneal chemotherapy (HIPEC) remains the cornerstone of treatment, offering improved survival outcomes by maximizing tumor debulking [5].

This report describes the case of a 75-year-old female who presented with diffuse abdominal pain, melena, and progressive weight loss. Initial diagnostic workup raised suspicion for a gastroesophageal (GE) malignancy. However, intraoperative findings revealed extensive mucinous peritoneal deposits in the absence of an identifiable primary lesion, and the diagnosis of PMP was established via histopathology. Given the rarity of such atypical presentations, this report underscores the importance of early suspicion, a comprehensive diagnostic approach, and multidisciplinary management. Integration of imaging advancements, pathological classification systems, and individualized treatment strategies is essential to improving outcomes in patients with elusive primary tumors.

## Case presentation

A 75-year-old female with no significant medical or surgical history presented with diffuse abdominal pain and melena for four days. She also reported a six-month history of anorexia, fatigue, and unintentional weight loss. There was no history of substance use, allergies, or familial illnesses. On physical examination, she appeared cachectic, with conjunctival pallor and a grossly distended, protuberant abdomen. A positive fluid thrill indicated the presence of ascites. Her vital signs were stable. There were no signs of jaundice, lymphadenopathy, or peripheral edema. Laboratory investigations (Table 1) revealed anemia, with a hemoglobin level of 7.5 g/dL, and an elevated erythrocyte sedimentation rate (ESR) of 135 mm/hour. Liver and renal function tests were within normal limits. Ascitic fluid analysis showed exudative fluid with a high protein content (5.8 g/dL), polymorphonuclear predominance, a low serum-ascites albumin gradient (SAAG: 0.1), and low adenosine deaminase levels (ADA: 3.0 U/L). Tumor markers, including CA-125, CA 19-9, and alpha-fetoprotein (AFP), were within normal limits. Carcinoembryonic antigen (CEA) was mildly elevated at 3.1 ng/mL.

**Table 1:** Key laboratory findings ADA: adenosine deaminase; CEA: carcinoembryonic antigen; ESR: erythrocyte sedimentation rate; Hb: hemoglobin; SAAG: serum-ascites albumin gradient

Parameter	Patient value	Reference range
Hematology		
Hb, g/dL	7.5	12.0–16.0
ESR, mm/hour	135	<20 (females)
Ascitic fluid analysis		
Total protein, g/dL	5.8	>2.5 (exudate)
SAAG, g/dL	0.1	<1.1 (exudative ascites)
Cell count (predominant cells)	Polymorphonuclear cells	Mixed in malignancy
ADA, U/L	3	<40
Tumor markers		
CEA, ng/mL	3.1	<3 (non-smoker)/<5 (smoker)

**Figure 1:**
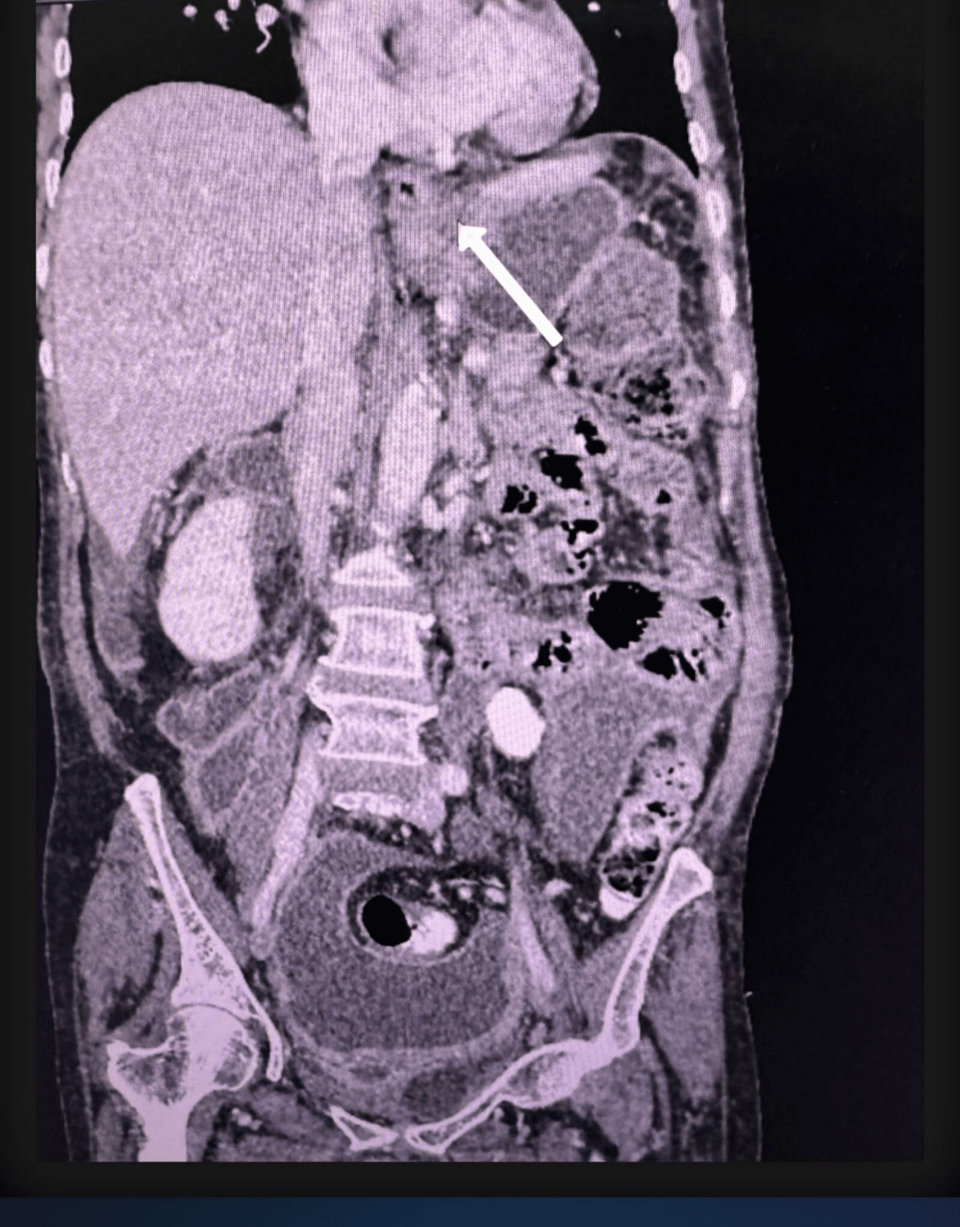
CECT axial section revealing the GE junction mass extending to cardia (arrow) CECT: contrast-enhanced computed tomography; GE: gastroesophageal

**Figure 2:**
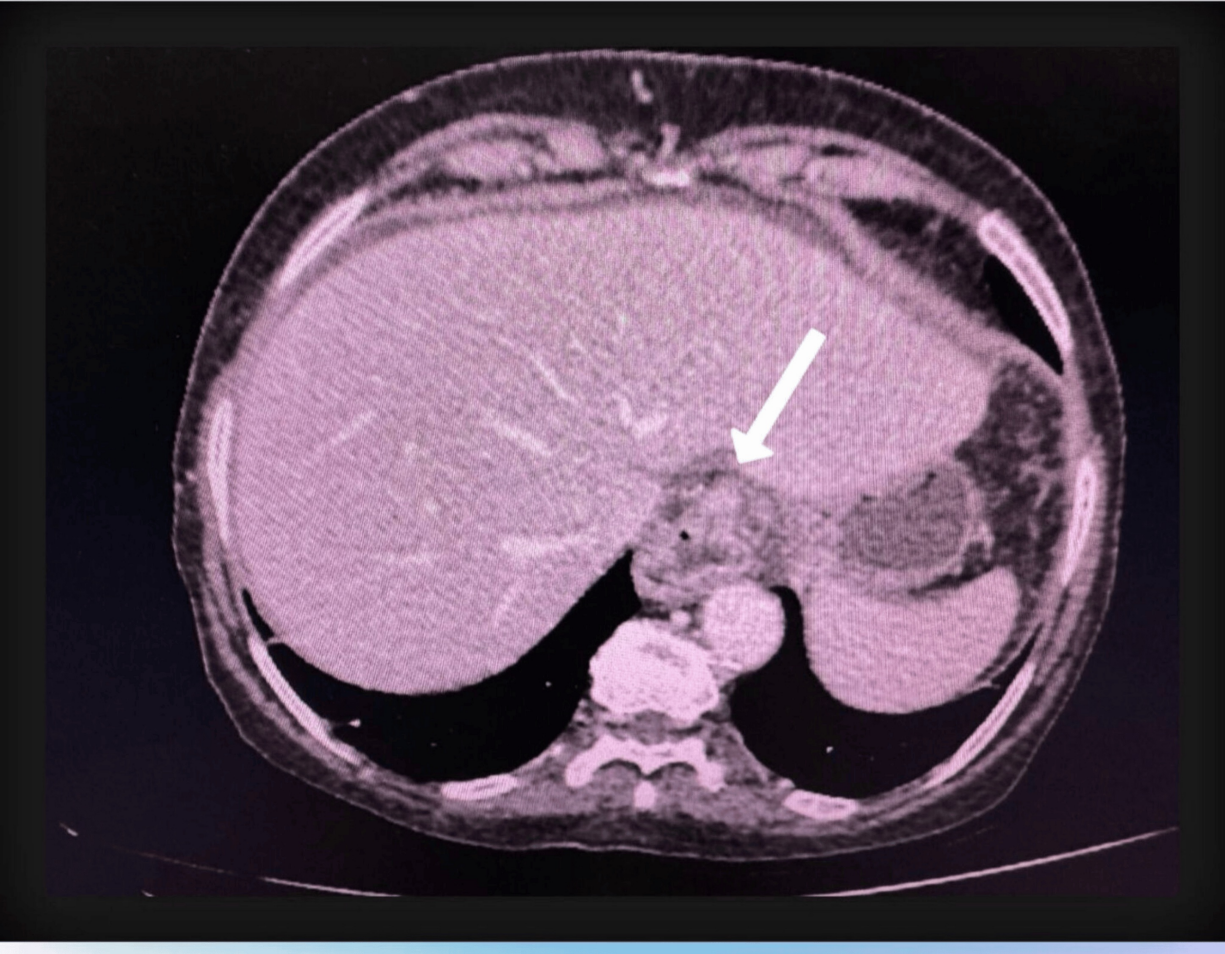
CECT showing irregular thickening of distal esophagus (arrow) CECT: contrast-enhanced computed tomography

CECT of the abdomen (Figures 1, 2) revealed a mildly dilated proximal esophagus with pooling of secretions and an irregular, heterogeneously enhancing thickening (7.6 × 2.0 cm) involving the distal esophagus and gastric cardia. Additionally, moderate to severe ascites and peritoneal thickening were noted. While a diagnostic ascitic tap was attempted initially, it yielded limited information regarding the primary pathology, and the patient underwent diagnostic exploratory laparotomy. Given the markedly distended abdomen, laparoscopy was deferred. Intraoperative findings revealed mucinous ascitic fluid and widespread peritoneal deposits. No primary lesions were identified in the appendix, ovaries, or elsewhere in the gastrointestinal tract.

**Figure 3:**
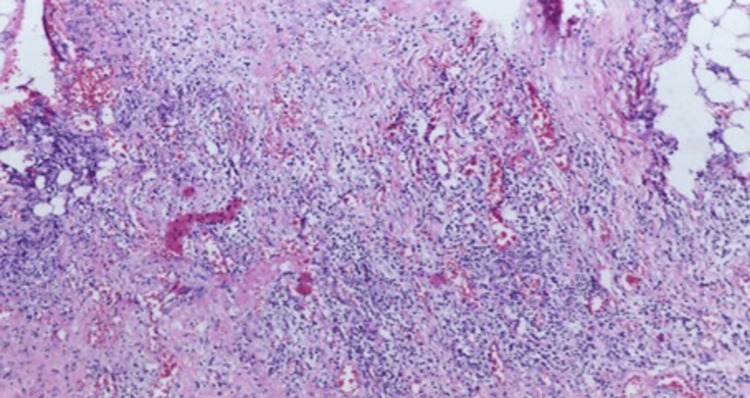
Lymphoplasmacytic infiltrate with adipocytes and congested blood vessels

**Figure 4:**
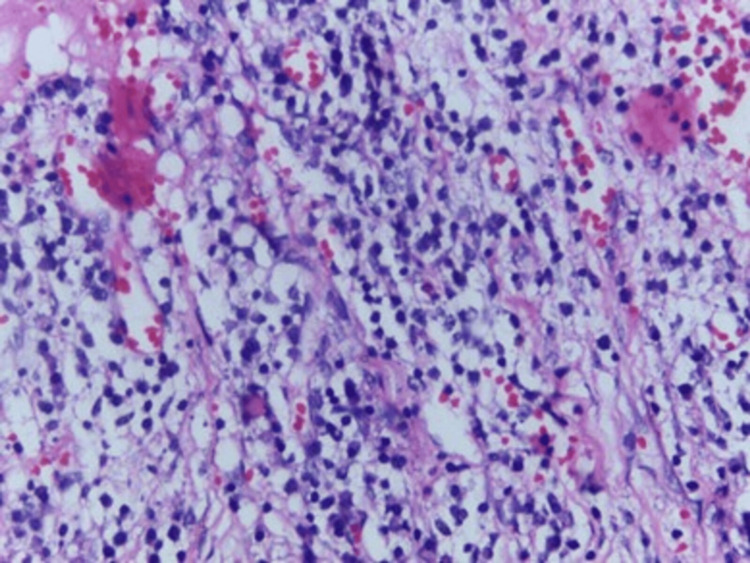
Lymphocytes and plasma cells forming the infiltrate

**Figure 5:**
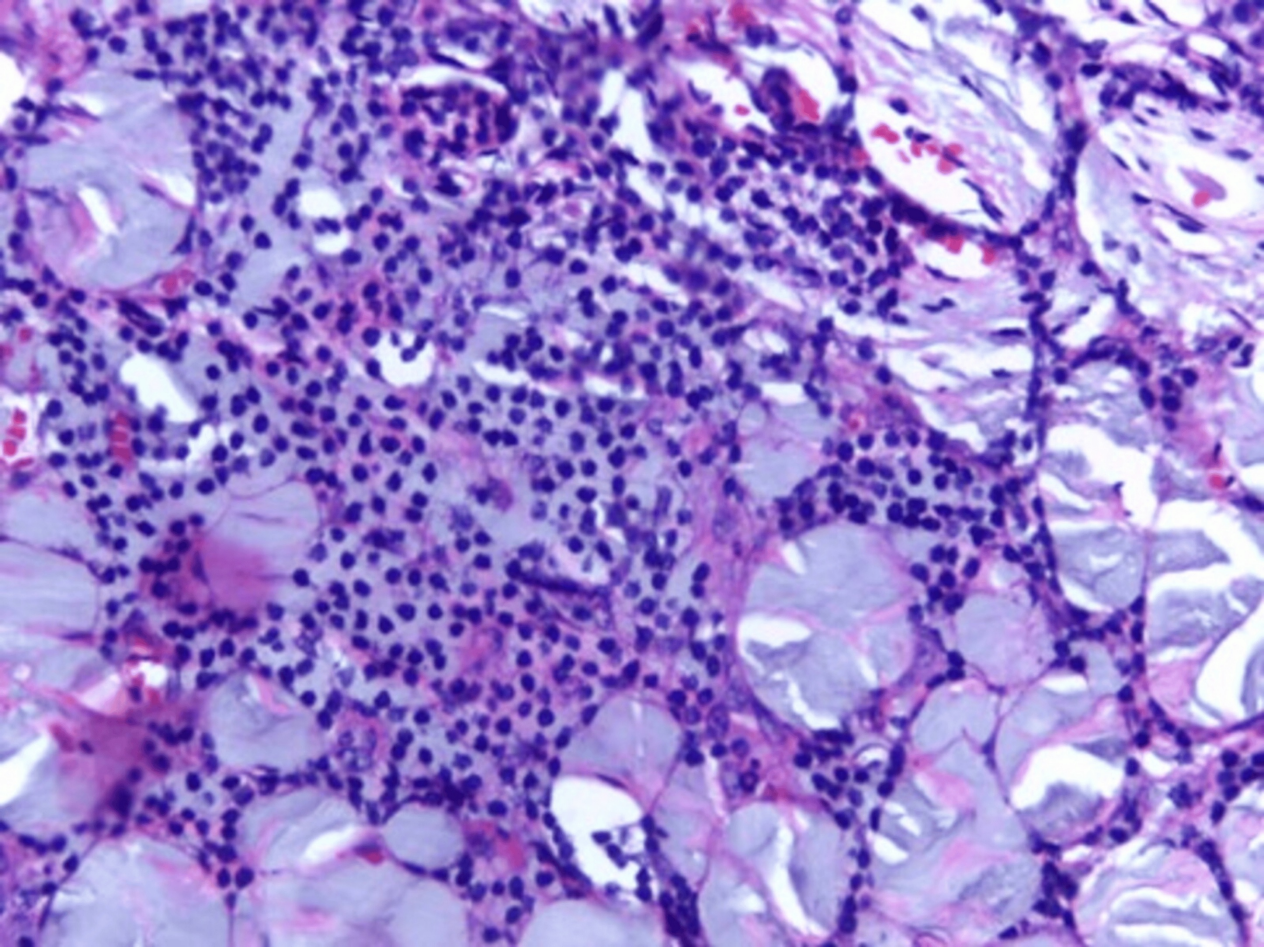
Focal aggregates of myxoid mucinous material were isolated in the background of lymphoplasmocytic inflitrate

Biopsies obtained from the omentum and peritoneum (Figures 3, 4, 5) showed myxoid stroma with pools of acellular mucin and chronic lymphoplasmacytic infiltration. No evidence of granulomas or epithelial malignancy was found. These findings were consistent with PMP. The suspected esophagogastric lesion could not be biopsied due to the patient’s declining performance status. Upper gastrointestinal endoscopy was not performed. The patient was stabilized and referred to a specialized oncology center for further evaluation.

Although the patient was referred to a higher center for further management, including potential CRS and HIPEC, she could not be initiated on definitive treatment. As per information received from the referral center, the patient succumbed to multiorgan dysfunction and sepsis before the initiation of HIPEC or cytoreduction.

## Discussion

PMP is a rare clinical condition characterized by mucinous ascites and mucin-producing epithelial cells dispersed across the peritoneal surfaces. Accurate diagnosis and timely management significantly influence patient survival and quality of life. Initially thought to originate from perforated appendiceal cystadenomas, PMP is now understood as the manifestation of disseminated mucin-producing neoplasms. While appendiceal tumors remain the most common source, other origins include the colon, rectum, stomach, pancreas, gallbladder, lungs, breast, fallopian tubes, and ovaries [2].

PMP is often diagnosed incidentally during abdominal surgery or imaging conducted for unrelated complaints, typically at advanced stages. A major diagnostic challenge is its nonspecific presentation, with many patients remaining asymptomatic or experiencing vague gastrointestinal symptoms. Misdiagnosis is common, and conditions such as irritable bowel syndrome are often considered initially [3]. Though more frequently observed in females, PMP has also been reported in male patients presenting atypically with symptoms such as nocturia, unexplained weight loss, or epigastric pain [4,5]. The clinical behavior of PMP varies widely, ranging from indolent to aggressive disease. The Peritoneal Surface Oncology Group International (PSOGI) classifies PMP into four histopathological subtypes: acellular mucin, low-grade mucinous carcinoma peritonei, high-grade mucinous carcinoma peritonei, and peritoneal mucinous carcinomatosis [6].

MRI of the chest, abdomen, and pelvis is the preferred imaging modality in suspected cases. However, CECT is preferred in the absence of MRI. A characteristic feature is “scalloping” of visceral surfaces, especially the liver and spleen, caused by mucinous loculations, which helps differentiate PMP from simple ascites [1]. However, primary tumors, especially appendiceal lesions, may be challenging to identify using CT. Gadolinium-enhanced MRI offers better soft tissue contrast in such cases, despite its relatively lower spatial resolution [5,7]. PMP remains exceedingly rare, with an estimated incidence of one to three cases per million people annually [7]. Due to its indolent nature, the disease is often not diagnosed until it is in its late stages. Reported five-year survival rates range from 53% to 75% [8].

CRS combined with HIPEC is the cornerstone of treatment and has improved 10-year survival rates to between 63% and 74% [9]. Despite often presenting as a benign-appearing, slowly progressive disease, PMP tends to recur and can be refractory to treatment. Surgical resection may include omentectomy, hemicolectomy, gastrectomy, or splenectomy, depending on disease extent. HIPEC involves intraperitoneal perfusion of heated chemotherapeutic agents such as mitomycin C, 5-fluorouracil, doxorubicin, irinotecan, or cisplatin [10]. However, upfront CRS and hyperthermic intraperitoneal chemotherapy (HIPEC) are generally not recommended for gastric or GE junction cancers with peritoneal metastasis. CRS may be considered following systemic chemotherapy in carefully selected patients who have limited peritoneal disease, typically indicated by a low PCI of less than 6. However, HIPEC is not considered the standard of care in these cases, as the evidence supporting its routine use remains limited and is still under investigation.

Colonoscopy can aid in preoperative assessment, particularly when appendiceal tumors spread to the colon. Without treatment, PMP can lead to massive ascites, bowel obstruction, or perforation [11]. Therefore, early suspicion and a structured diagnostic approach are essential. Similarly, GE junction tumors arise at the transition between the distal esophagus and proximal stomach. The majority are adenocarcinomas and are classified by the Siewert system into three types: type I (distal esophageal), type II (true cardia), and type III (subcardial) tumors [12]. Risk factors for GE junction tumors include alcohol consumption, smoking, chronic GE reflux disease (GERD), and conditions such as achalasia. These tumors, like PMP, are often asymptomatic in early stages and typically present late, with odynophagia, weight loss, malnutrition, or aspiration pneumonia due to regurgitation [13]. Diagnostic tools such as barium swallow and upper gastrointestinal endoscopy are crucial in evaluating patients with dysphagia. Surgical resection, including esophagectomy and lymphadenectomy, remains the primary curative option and offers long-term survival in about one-third of cases [14].

In summary, both PMP and GE junction tumors are insidious in onset and frequently diagnosed in advanced stages. High clinical suspicion, timely diagnostic imaging, histological evaluation, and aggressive surgical management are essential to improving patient outcomes and overall survival.

## Conclusions

PMP is a rare peritoneal malignancy that often presents with vague symptoms and is diagnosed at an advanced stage. In our case, extensive mucinous ascites and peritoneal deposits were identified, but no clear primary lesion was found. Despite histopathological confirmation, the patient’s condition deteriorated rapidly, and she succumbed to multiorgan dysfunction before CRS or HIPEC could be initiated. This report highlights the need for early clinical suspicion, especially in patients with unexplained ascites and weight loss. When imaging and fluid analysis are inconclusive, exploratory surgery may be required. Histopathology remains essential for diagnosis, and timely referral to specialized centers is critical. A multidisciplinary approach, rapid decision-making, and awareness of atypical presentations can significantly influence outcomes in PMP.
